# Neutrophil Extracellular Traps (NETs) and Damage-Associated Molecular Patterns (DAMPs): Two Potential Targets for COVID-19 Treatment

**DOI:** 10.1155/2020/7527953

**Published:** 2020-07-16

**Authors:** Sebastiano Cicco, Gerolamo Cicco, Vito Racanelli, Angelo Vacca

**Affiliations:** Department of Biomedical Sciences and Human Oncology, University of Bari Aldo Moro Medical School, Piazza G. Cesare 11, I-70124 Bari, Italy

## Abstract

COVID-19 is a pandemic disease caused by the new coronavirus SARS-CoV-2 that mostly affects the respiratory system. The consequent inflammation is not able to clear viruses. The persistent excessive inflammatory response can build up a clinical picture that is very difficult to manage and potentially fatal. Modulating the immune response plays a key role in fighting the disease. One of the main defence systems is the activation of neutrophils that release neutrophil extracellular traps (NETs) under the stimulus of autophagy. Various molecules can induce NETosis and autophagy; some potent activators are damage-associated molecular patterns (DAMPs) and, in particular, the high-mobility group box 1 (HMGB1). This molecule is released by damaged lung cells and can induce a robust innate immunity response. The increase in HMGB1 and NETosis could lead to sustained inflammation due to SARS-CoV-2 infection. Therefore, blocking these molecules might be useful in COVID-19 treatment and should be further studied in the context of targeted therapy.

## 1. Introduction

COVID-19 is a pandemic challenge caused by the new coronavirus SARS-CoV-2 [1] that is currently imposing heavy stress on many health systems worldwide. It belongs to the coronavirus family that includes the Severe Acute Respiratory Syndrome Coronavirus type 1 (SARS-CoV) and Middle East Respiratory Syndrome (MERS-CoV) viruses. Coronaviruses have a preferential tropism for lung cells [2]. SARS-CoV-2 is known to use the same receptor as SARS-CoV to enter the host cell, namely, angiotensin-converting enzyme II (ACE2) [2]. Acute SARS-CoV-2 patients present with a wide range of clinical manifestations, ranging from asymptomatic or mildly symptomatic (common cold) up to severe, often fatal disease. The latter form usually presents with bilateral interstitial pneumonia and moderate to severe oxygen desaturation and hypoxia. Many patients develop respiratory failure (RF) and acute respiratory distress syndrome (ARDS) [3], requiring prompt admission to the intensive care unit (ICU). Unlike the usual ARDS, these patients show a normal or slightly increased lung compliance and mostly need high-flow oxygen or continuous positive airway pressure (CPAP) ventilation [4]. The ventilation outcome of SARS-CoV-2 pneumonia is similar to the one described in the respiratory failure in interstitial lung disease [5].

SARS-CoV-2 raises many immunological questions. Reports [6] and Chinese guidelines [7] have identified alveolar damage. Previous reports based on viruses of the same family indicate a cytokine storm. The first Chinese report identifies an increase of IL-6 in these patients [8] that peaks in severe cases. The main treatment strategy is based on cytokine blockade to modulate inflammation. Some of the drugs most commonly used to treat SARS-CoV-2 in off-label indications are chloroquine (CQ) and/or hydroxychloroquine (HCQ). These drugs exert multiple anti-inflammatory effects and are well known to be effective in treating chronic inflammatory diseases such as lupus and rheumatoid arthritis. The anti-inflammatory mechanisms are not fully understood. However, it has been established that they are able to block autophagy, interfering with DNA repair and lysosome formation by elevating vacuolar pH [9, 10]. CQ/HCQ also reduces neutrophil extracellular traps (NETs), as well as the secretion of damage-associated molecular patterns (DAMPs) [11]. Recent data from COVID-19 autopsies described neutrophil infiltration in the lung airspace [12] and blood vessels [13]. Moreover, compared to those of healthy volunteers, in COVID-19 blood samples, Zuo et al. found increased NETs, quantified as cell-free DNA, myeloperoxidase- (MPO-) DNA, and citrullinated histone H3 (Cit-H3) [14], that were correlated with clinical biomarkers. The clinical presentation seems to be a consequence of DAMP action on the immune system. From clinical data on COVID-19 and empirical data on CQ/HCQ use, it could be speculated that these two mechanisms may be key players in immune modulation and SARS-CoV-2 infection host damage. This review focuses on the possible role of NETs and DAMPs in lung damage due to SARS-CoV-2 infection, making immunological suggestions about possible disease treatment targets.

## 2. Neutrophil Extracellular Traps (NETs) and Respiratory Virus Infection

NETs are large extracellular, web-like structures released from neutrophils in the extracellular space. They are one of the weapons in the neutrophil arsenal employed to fight pathogens. These structures are composed of decondensed chromatin and cytosolic and granule proteins [15]. The DNA in NET derives from the nucleus and mitochondrial material. Two forms of NET are known. One is suicidal NETosis. It is a several-hour time-frame process in which neutrophils decondense their nuclear chromatin and DNA in the cytoplasm. Next, chromatin and DNA mix with granule-derived antimicrobial peptides. Finally, this mixture is released into the extracellular space with a spread of Reactive Oxygen Species (ROS) [16]. The second form is vital NETosis where NETs are released without cell death; thus, cells are able to survive and are still capable of normal functions including phagocytosis. Unlike suicidal NETosis, vital NETosis does not require the generation of ROS nor the activation of the Raf/MERK/ERK pathway and occurs quickly, usually within 5 to 60 min after cells are stimulated [17, 18]. The neutrophil stimulation occurs via toll-like receptor (TLR) or complement receptor for C3 protein ligand binding. The activation of these pathways induces a change in nuclear membrane morphology. Vesicle budding starts. Hence, vesicles containing nuclear DNA move through the cytoplasm, coalesce with the plasma membrane, and release their load extracellularly [17–19].

NETs are useful to prevent the dissemination of pathogens, thanks to their neutralizing and killing functions [20]. Of note, while NETosis is directly induced by extracellular fungal hyphae, large bacteria and their aggregates [20], intracellular bacteria, are not able to form NETs [20].

For small pathogens like viruses, NETs play a double-edged role. One mechanism is virus entrapping by NETs, as observed in syncytial respiratory virus (RSV) infection [21] or influenza [22], but no antiviral role has been found *in vivo* [23]. Both these viruses, like SARS-CoV-2, are RNA viruses, causing 3–5 million severe cases and 250,000– 500,000 deaths worldwide per year [24]. They replicate in respiratory epithelial cells and cause necrotic tissue damage. In influenza virus infection, a great increase in NET gene activation and expression has been found but there is no increase in cytokine production [25]. This relates to a mode of neutrophil activation by a virus depending on the disease severity [25]. In fact, different NETosis forms according to the disease severity: in mild influenza, no NET formation occurs [26], while in severe influenza [27] and lethal disease [28], NETs do commonly form. Moreover, in virus infection, they are effective to block viruses at the infection site, entrapping them in a DNA web. Virus exposure to other immune cells might achieve cleavage by macrophages [29].

At the same time, an increase in blood neutrophils has been described in people who died of influenza pneumonia [27]. The NET-associated antimicrobial factors could be detrimental for the host. Antimicrobial proteins released with NETs may be directly toxic to tissues, and their massive production may produce tissue damage [30]. Indeed, NET formation releases elastase and myeloperoxidase that are useful to not only cleave host proteins at the site of infection but also produce tissue injury [31, 32].

NET formation depends on TLR-4 activation, and NETosis is triggered by the virus proteins binding to TLR-4 [21]. In fact, RSV fusion proteins are able to induce neutrophil DNA release in the extracellular space due to TLR-4 signaling activation. This mediates NETosis via a signal transduction cascade that activates the assembly of NADPH oxidase complex and ROS formation [33]. By using neutralizing antibodies against TLR-4, the extracellular DNA production is profoundly inhibited [21]. Coronavirus fusion proteins may possibly induce a similar mechanism [34, 35].

During acute lung inflammation following influenza virus infection, an excessive infiltration of neutrophils takes place in the lungs [22]. However, neutrophils play both protective and detrimental roles in the lungs [22, 36]. These cells produce excessive NETs in response to the influenza A H1N1 virus. NET formation is dependent on histone deamination by Protein Arginine Deaminase 4 (PAD4) [23]. In NETs, *α*-defensin-1, an antimicrobial protein able to directly inhibit the H1N1 virus replication by blocking protein kinase C (PKC) in infected cells, is also present [37]. The *α*-defensins increase during the H1N1 virus infection [38] and inactivate the virions sequestered in NET fibres, thus preventing them from entering the target cells in the lungs. But the *α*-defensins are also able to inflict damage to host cells and tissues due to their cytotoxic properties [36].

Also, in the lungs, NET proteins cause capillary destruction and leakage and induce endothelial cell death and thrombosis as well as lung epithelial destruction and death [39–41]. On the other hand, NETosis is directly induced by lung epithelia via damage-associated molecular patterns (DAMPs) following infection by the influenza virus [27]. These phenomena ultimately lead to the evolution of the infection, so neutrophil activation and NET formation are predictors of respiratory failure (RF) and acute respiratory distress syndrome (ARDS) [25, 27, 40]. The detrimental role of NETs might be related to prolonged exposure to a virus. It could be assumed that NET formation is the first line of defence against viruses. As the same occurs in influenza and syncytial viruses, small pathogen sizes lead to an excessive NETosis. The net result is an accumulation of activated neutrophils that release molecules that are toxic for host tissues.

It has also to be considered that NETosis increases in different settings. In fact, these differences are able to influence NET formation. In fact, NET increases are related to a fatty diet [42], obesity [43, 44], diabetes [45, 46], age [47], and sex differences [48, 49]. Worthy of note is that in females, NETosis activation is reduced due to progesterone release [50].

Pathological [6], biochemical [8, 51], and clinical [1, 3, 51] findings in SARS-CoV-2 infection appear to be related to the NET reaction induced by the lung epithelium damage and disease severity. Previous findings in other virus lung injury as well as the data on serum NET increases in COVID-19, the cytokine profile and clinical evolution, support the idea that NETs are the main actors in severe pneumonia related to SAR-CoV-2 infection. Patients experience a clinical evolution of COVID-19 from a first asymptomatic or mildly symptomatic period up to severe pneumonia. NETs had been found to be increased in COVID-19 compared to healthy controls, but they also resulted increase in ventilated patients compared to those who did not undergo ventilation [14]. These data underline the possibility that NETs may be the key player in the clinical evolution as a result of continual strong stimuli to their formation in COVID-19. This mechanism may be related to different factors. Firstly, the fast spread of the virus will induce a rapid increase in viral load in lung tissue. As discussed above, viral proteins might be considered potent activators of NETosis. Moreover, damaged tissues and neutrophils themselves both produce cytokines that enhance neutrophil activity. In fact, cytokines result are strongly produced after SARS-CoV-2 stimulation. Their action will play a role in the chemotactic recruitment of neutrophils, enhancing ROS and NET formation. The induction of NETosis is also due to other molecules secreted by damaged tissues such as damage-associated molecular patterns (DAMPs).

## 3. Damage-Associated Molecular Patterns (DAMPs) and Virus Infection

Cells release DAMPs as endogenous danger signals that alert the innate immune system to unscheduled cell death following microbial invasion or stressors [52]. These endogenous self-antigens, such as high-mobility group box 1 (HMGB1) and heat shock proteins (HSPs), activate signaling of mitogen-activated protein kinases (MAPKs) and nuclear factor of kappa light polypeptide gene enhancer in B-cells (NF-*κ*B), which trigger the inflammatory response [52, 53]. HMGB1 is a chromatin-associated protein formed by two domains connected by a nine-amino acid loop and a highly disordered negatively charged C-terminal tail [54, 55]. It performs different functions depending on its cellular localization. As a nuclear protein, it is involved in DNA repair, transcription, and genome stability [55–57], while during cellular death or inflammation, it is released into the extracellular space where it is classified as an alarmin [58, 59]. It induces the release of inflammatory cytokines such as tumour necrosis factor (TNF), interleukin- (IL-) 1*β*, and IL-12 that aggravate acute tissue damage.

DAMPs are involved in the pathways of both NETosis and autophagy [53]. The nuclear and cytosolic environments are characterized by a negative redox potential that maintains HMGB1 in a fully reduced form (fr-HMGB1). This form binds the receptor for advanced glycation end products (RAGE), a receptor that is constitutively highly expressed on lung alveolar epithelial cells but has little or no expression during basic conditions in other tissues [60].

The HMGB1-RAGE interaction triggers neutrophil-mediated injury amplification following necrosis [61]. Recently, it has been demonstrated that RAGE mediates HMGB1 endocytosis to the endolysosomal compartment [62, 63].

This pathway is an important one that alerts cells to a dangerous extracellular environment. HMGB1 acts as a detergent in the lysosomal membrane due to the acidic conditions inside the lysosome system [63, 64]. The HMGB1-transported partner molecules avoid the expected lysosome degradation and leak into the cytosol to reach their cytoplasmic receptors, stimulating an inflammatory response. HMGB1-RAGE is also an important pathway regulated by autophagy. In fact, in *atg7* autophagy gene-deficient mice, the HMGB1-RAGE axis promotes macrophage activation in a paracrine loop [65].

Biological implications of this mechanism may be fundamental in the pathogenesis of severe pulmonary inflammation due to high constitutive cell surface RAGE expression. An increase in HMGB1 release has been observed during acute lung injury (ALI) under proinflammatory stimulation [66], but in the same model, autophagy stimulation was effective in reducing HMGB1 release and ALI. In fact, in preclinical and clinical studies, it has been demonstrated that RSV generates extracellular HMGB1 release in pulmonary inflammation and that HMGB1-specific antagonists ameliorate these conditions [67, 68].

During inflammation, the extracellular space with a rich ROS content leads to the formation of a disulphide bond- (ds-) HMGB1 that activates TLR-2 and TLR-4, inducing the release of proinflammatory chemokines and cytokines that activate both innate and adaptive immunities [69, 70]. CXC ligand 12 (CXCL12) is expressed in many tissues under both homeostatic and inflammatory conditions and can stimulate cellular recruitment by activating the CXC chemokine receptor type 4 (CXCR4) [71]. The heterocomplex formed by CXCL12/HMGB1 plays a pivotal role in the pathogenesis of many inflammatory processes such as rheumatoid arthritis [72], mainly promoting the migration of monocytes [73]. CXCL12 binds HMGB1 only as fr-HMGB1 [74]. The heterocomplex formation occurs in the extracellular space where ROS lead to ds-HMGB1 rather than fr-HMGB1. To avoid this event, cells may release glutathione reductase and enzymes of the thioredoxin system against the ROS, thus preserving the fr-HMGB1 form [72, 74, 75] and its function in inflammation and sepsis. In acute lung diseases, HMGB1 acts as an activator of innate immunity, leading to the production of IL-1*β*, IL-6, and TNF-*α*. Sepsis or ARDS triggers HMGB1, thus strongly activating the innate immunity. However, HMGB1 also induces NET formation [76]. As explained in the previous section, HMGB1-induced NETosis may also cause tissue damage. In a recent study, HMGB1 in LPS-induced acute lung injury caused proinflammatory polarization of macrophages through the binding of TLR-2 and TLR-4 which stimulate the protein kinase R (PKR) [77]. Large amounts of TLR-4 have been found in males, consequent to testosterone production [78]. Testosterone induces DAMP release resulting in increased TLR4 signaling in males compared to females. The final result is a higher proinflammatory cytokines (like IL-1*β* and IL-18) production in males compared to females. In COVID-19 patients, an increase in many cytokines has been found, including IL-6 and IL-8 [8], especially in severe cases. These cytokines are also increased in lung injury. In an experimental *in vivo* model, it has been demonstrated that an increase in HMGB1 may be mediated by autophagy [79]. HMGB1 also stimulates the production of IL8 [80] a cytokine that plays an important role as a chemoattractant in the activation of the PI3K/Akt/mTOR pathway [81]. In fact, rapamycin-treated mice present reduced lung damage compared to vehicle-treated counterparts. However, this effect did not appear to be related to immune suppression, because rapamycin did not influence the IL-6 production [79]. IL-6 is also increased in males compared to females contracting influenza A pneumonia [82].

This is in line with epidemiological data about the greater severity of SARS-CoV-2 disease in males than in females [3, 83]. Consistent with a previous literature, the IL-6 increase in COVID-19 severe patients [8] should be related to HMGB1 macrophage release [80], but further investigations are needed. The link between HMGB1 and TLR-4 also acts as a procoagulant factor through platelet stimulation of this receptor [84]. Some aspects of SARS-CoV-2 disease overlap those of flu, especially the excessive activation of innate immunity. Nosaka et al. showed that mice with H1N1 disease treated with an anti-HMGB1 mAb were protected against the virus and showed a longer overall survival than the control group (1 death among 15 mice in the anti-HMGB1 group *versus* 8 of 15 in the control group) [85].

**Figure 1:**
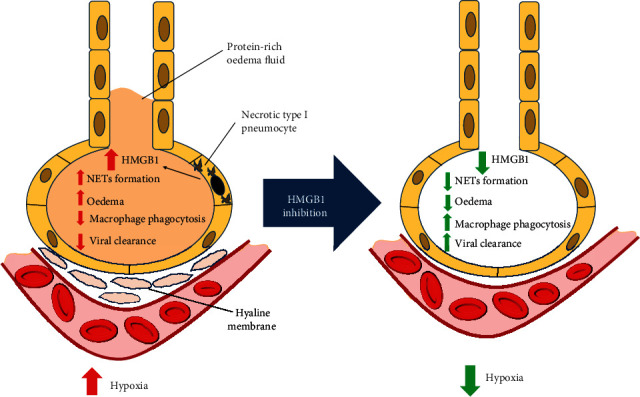
Effects of HMGB1. Necrotic lung epithelium releases HMGB1, whose increase induces pulmonary oedema, macrophage phagocytosis, and NETosis, reducing viral clearance and leading to hypoxia. HMGB1 blockade may reverse these effects, influencing the reduction of hypoxia. HMGB1: high-mobility group box 1; NETosis: release of neutrophil extracellular traps.

Recently, the use of anti-HMGB1 mAb in conjunction with neuraminidase inhibitor Peramivir® has demonstrated an excellent therapeutic activity in mice with the H1N1 disease [86]. This could also be explained by increased virus clearance [87]. Entezari et al. [88] used an anti-HMGB1 mAb in mice with cystic fibrosis, obtaining a reduction in pulmonary oedema and lung injury and an enhanced macrophage phagocytosis against *Pseudomonas aeruginosa*. Data on patients affected by lung infection due to a combination of influenza virus and bacteria showed an increase in serum concentrations of HMGB1 and IL-6 [89]. These data suggest the importance of anti-HMGB1 treatment even during bacterial superinfections in patients with SARS-CoV-2 (Figure 1). In fact, HMGB1 may play a key role in promoting and sustaining lung injury. However, the difference in SARS-CoV-2 compared to the other respiratory viruses must be noted. In fact, recent data indicate a greater role of this virus in causing pulmonary vascular damage compared to influenza virus [90]. Vascular damage is one of the key factors promoting tissue hypoxia. The lung capillary thrombosis found in COVID-19 lung specimens increases the dead space, inducing ischemic injury with ROS production. ROS are also crucial molecules in HMGB1 activation. In fact, hypoxia [53] activates HMGB1 leading to lung damage via autophagy and NET activation. Similarly, hyperoxia lung damage is attenuated in an *in vivo* mouse model if HMGB1 is previously blocked by specific antibodies [91]. The finding of increased NETs in ventilated severe COVID-19 patients [14] may be both a consequence and a trigger of HMGB1 release in a dangerous self-perpetuating loop. Hypoxia may induce cell death resulting in increased extracellular HMBG1. At the same time, ROS induce NETs and autophagy. The latter mechanisms combined with DAMP release are able to sustain a robust inflammatory response, enhancing leukocyte attraction and tissue infiltration. Activated immune cells participate in this process via a paracrine release of multiple cytokines starting from IL-6 that is ubiquitously released during inflammation and infections. All these pathways should result in host tissue damage and, finally, in a great clinical severity of SARS-CoV-2 infection, possibly inducing lung failure and death.

## 4. Interleukin-6, NETs, and Viruses

The IL-6 family is a group of cytokines that use the common signaling receptor subunit glycoprotein 130 kDa (gp130) [92]. It comprises a large number of molecules carrying out numerous cell functions, including B-cell stimulation and the induction of acute phase proteins as well as metabolic and neurotrophic functions [92]. Viruses do not appear to be strong inducers of IL-6 per se [93]. In fact, IL-6 is largely induced by large size pathogen infections [94] in which it strongly stimulates acute phase protein production. IL-6 upregulation is a key factor in priming naïve T-cells for T helper lymphocyte (Th)17 differentiation, inhibition of the immunosuppressive functions of regulatory T-cells (Tregs), and the prevention of Th17 cell conversion into Tregs [93, 95]. The Th17 response is characterized by the production and release of IL-17 and IL-22 that enhance mucosal barrier function through the expression of antimicrobial peptides and neutrophil recruitment [95, 96]. Th17 responses may contribute to viral persistence due to virus upregulation of antiapoptotic molecules, blocking target cell destruction by cytotoxic T lymphocytes (CTL) and enhancing the survival of infected cells [97]. Thus, the Th17 response may be very effective to block killing intracellular pathogens such as intracellular bacteria and viruses in the setting of a strong Th1 response via the release of interferon- (IFN-) *γ* [98]. To obtain the Th17 responses, IL-1*β*, IL-12, TNF-*α*, and IL-6 are produced by dendritic cells (DCs) under TLR stimuli [99–101], to induce Th1 polarization in T lymphocytes [102]. Cytokine DC production is also modulated by HMGB1. In the neutrophilic asthmatic model, HMGB1 modulates Th17 differentiation, stimulating proinflammatory cytokine production released by DCs [103]. The authors also demonstrated a reduction of Th17 activation via blockade of activated DCs with a specific HMGB1 inhibitor. Also in influenza infection, HMGB1 may induce IL-6 and IL-8 production, activating DCs via the TLR-4 pathway [104]. In this *in* vivo model of influenza infection, the use of the specific inhibitor of TLR-4 stimulation of human DCs resulted in the reduction of influenza virus-related lethality, proinflammatory cytokine gene expression in the lungs, and acute lung injury (ALI). This effect is induced by reducing the TLR-4-dependent cytokine storm mediated by HMGB1 [104].

Literature data indicate that during aging, Th1 shifts to the Th2 cytokine response [105]. Th2 lymphocytes are necessary to regulate inflammatory responses and are increased in asthma and autoimmune diseases. In older people, TLR-induced Th1 cell differentiation stimulates antigen-specific CTL activation after vaccination, resulting in an increased response to the vaccine, that contributes to an improved clinical protection against severe diseases when antibodies fail to provide sterilizing immunity and prevent infection [98, 105, 106]. In particular, it has been demonstrated that TLR-4 is a crucial pathway for IL-6 production in the elderly. In fact, in older people, DCs are an important source of IL-6, showing higher levels than in young people after TLR-4 stimulation via a specific agonist [107]. Thus, in the elderly, there is a significant increase in IL-6 production as a consequence of antigen stimulation as compared to young people [107]. The increased mortality and severity of COVID-19 in the elderly may be the consequence of the immunological shift from Th1 to Th2. This response was shown to be ineffective against viral infection, so DC cytokine production sustained it via a Th17 response.

In an *in vivo* model, hypoxia induces an increase of HMGB1, TLR-2 and TLR-4, and IL-6, IL-8, and IL-10 in the respiratory system and blood [108]. At the same time, the production of IL-6 and other inflammatory cytokines also increases, as a consequence of HMGB1 stimulation by hypoxia in both lung tissue and alveolar macrophages [53]. These stimuli are not effective for a strong increase of acute phase proteins such as C-reactive protein (CRP) or Procalcitonin (PCT). In fact, unlike in bacterial infections, in viral infection, there is little increase of CRP and PCT, likely due to an increased IFN-*γ* production as a result of IL-6 and TNF-*α* stimulating Th1 differentiation [94]. The significant IL-6 increase seen during hypoxia in SARS-CoV-2 patients [109] is consistent with these data. The detrimental role in IL-6 function is mostly due to the host lung damage induced. Immune infiltration, rather than effectively obtaining viral cleavage, tries to block virions by entrapping them in NETs. This results in an increase of infective cell infiltration. SARS-CoV-2-induced hypoxia and tissue infiltration induce cell necrosis and HMGB1 release, promoting DC IL-6 production in an exponential enhancement of the immune response.

IL-6 has been found to be increased in obese people, the elderly, and males. Obese patients present a basal inflammatory state [110]. It has been demonstrated that the increased levels of IL-6, IL-8, and TNF-*α* in obesity are due to adipose tissue release [111]. The inflammatory cytokines are also related to insulin resistance in obesity [111, 112]. IL-6 increases during life, being higher in the elderly compared to young people [113, 114], since older people present a low-grade inflammatory state related to the development of the principal chronic degenerative diseases. Sex also influences the cytokine profile. In fact, males have an increased production of IL-6 in basal conditions, while in women there is a strong increase in cytokine production during infections [113]. On the contrary, males present persistently increased IL-6 during hypoxia in haemorrhagic shock [115]. Senescence of the human body may be the cause and consequence of increased IL-6. Aniszewska et al. [116] demonstrated that IL-6-deficient mice are more active than wild-type mice. In another study, compared to nonfrail patients, IL-6 results in increase in frail patients and are inversely related to haemoglobin [116].

Besides the host characteristics, increases in IL-6 and other cytokines production are mostly related to the virus pathogenic power. In fact, it has been demonstrated that highly pathogenic influenza viruses enhance the production of IL-6 and TNF-*α* compared to low pathogenic viruses [22]. Indeed, an active virus replication in human macrophages and DCs has been described [22], and during this process, both NETs and HMGB1 may play a role. In fact, Sabbione et al. [117] found an increased expression of IL-6 and IL-8 as a consequence of NET stimulation of lung cells. The expression of both cytokines was increased in the presence of septic antigens and HMGB1. The authors also found an increased cytokine expression if NETs were stimulated by smoke extracts, mimicking what happens in smokers in lung cell/NET interaction [117]. This is consistent with the finding that active smoking is correlated with an increased severity of COVID-19 [118]. It could be speculated that this result may be a consequence of increased ROS and hypoxia experienced in smoker patients.

Coronaviruses may account for all these effects in COVID-19. In fact, IL-6 and TNF-*α* expression results are modified in an *in vivo* diabetic model, in particular as an early response to MERS-CoV infection [119]. All this evidence could explain the risk of severe disease in SARS-CoV-2 [8, 51, 83] depending on the increase of IL-6, while the polarization to Th2 in diabetes should be a consequence of impaired cytokine production. Moreover, preprint indicates that IL-6 levels in SARS-CoV-2 infection result are inversely related to T-cell reduction [120]. COVID-19 may finally cause a cytokine storm [121]. The virus induces HMGB1 release and NETosis in the attempt to obtain virus cleavage. The ineffective result leads to immune system dysfunction, due to an excessive Th17-Th2 response, associated to a strong cytokine release by DCs, which may induce severe COVID-19 disease. IL-6 is one of the leading cytokines in this process. Host characteristics, like the male sex, obesity, frailty, and advanced age, are cofactors that enhance the storm. The prevention of the cytokine storm may be imperative in COVID-19 management, but medications and ventilation also influence the cytokine release. In this scenario, the regulation of autophagy might be considered in order to modulate the immune response.

## 5. Autophagy, Immunity, and Virus Infection

Autophagy is a survival mechanism used by cells when energy supplies are low, recovering nutrients by digesting their own metabolic waste [122]. After encapsulating the metabolic waste, the bilayer membrane gradually collapses to form a closed autophagosome before it fuses with lysosomes, obtaining an autolysosome [123]. Digested contents are released into the cytoplasm to be used in biosynthesis. This mechanism is induced by *Atg* genes [123] and regulated by numerous intracellular signaling pathways, including the AMPK, PI3K/Akt, and MAPK/ERK pathways that intersect at the mammalian target of rapamycin (mTOR), which is a negative regulator of autophagy [122, 124]. Autophagy plays an important role in immune response and cell death. Cells use this mechanism to resist pathogen invasion. Autophagy participates in both the innate and adaptive immune responses [125, 126]. However, an excessive immune response and the production of excess antibodies mediated by autophagy can also cause tissue damage and induce autoimmune disease [127].

Cell death characterized by NET formation requires both autophagy and ROS generation [123, 128]. Cheng et al. [129] proved that autophagy and ROS are crucial for mitogen phorbol myristate acetate- (PMA-) induced NETosis, but ROS can induce NETosis independently of autophagy. Autophagy-driven NETosis is also regulated by mTOR. Using rapamycin, a pharmacological inhibitor of the mTOR pathway, both increased autophagy and accelerated NETosis due to hypoxia-inducible factor 1*α* (HIF-1*α*) activity which was demonstrated [130]. Thus, hypoxia due to HIF-1*α* activity is an important inducer of NETosis.

Cytokines also induce autophagy and NETosis. Pham et al. [131] demonstrated that IL-8 strongly induces autophagy and NETs in neutrophils, in which they were reciprocally correlated. Autophagy not only does actively participate in NET formation but also inhibits the excessive release of NETs. A study of mice infected with *Pseudomonas aeruginosa* and affected by pneumonia showed that the activation of innate immunity improves the disease outcome via antimicrobial agents that enhance autophagy to rapidly eliminate NETs, thus preventing severe tissue damage [132]. Enhancement of NET generation and release, which requires autophagy, is the key process in antimicrobial treatment, providing evidence of the dual role of autophagy in NETosis.

Studies showed that the use of final period autophagy inhibitors, such as bafilomycin A1 (Baf-A1) and chloroquine (CQ), decreases NET production following a reduction of autophagy in a septic setting [133, 134].

As stated above, NETs are beneficial to their host in response to infection, but in the absence of autophagy, an excessive production of NETs can cause severe tissue damage [27, 36, 39]. This is particularly true in the damage mediated by autophagy [123].

SARS-CoV-2 induces thrombus formation, demonstrated especially in lung specimens [6, 13, 90]. This pathological finding may be explained by autophagy activation and NET formation. Manfredi et al. [135] provided evidence that autophagy is always accompanied by the generation and release of NETs in inflammatory disease. They observed that the use of inhibitors of autophagy flux prevents the generation of NETs. Similarly, they found that neutrophils pretreated with low molecular weight heparin *in vitro* show a significantly reduced autophagy activity and excess of NETosis, even if cells were stimulated by IL-8 or HMGB1. The latter molecule has been indicated to stimulate autophagy and NET formation by activating platelets, while none of these mechanisms was induced by *hmgb1* gene-deficient platelets [84]. Finally, similar significant anti-inflammatory results were found in healthy volunteers, following a single prophylactic dose of parnaparin. Another study showed that the antiviral cytokine IFN-*λ*1/IL-29 could reverse the formation of NETs induced by inorganic polyphosphates (polyP), due to counteracting the inhibitory effects of polyP on mTOR, via a decrease of autophagy [136].

Studies on autoinflammatory and autoimmune diseases indicate a regulation role in the development and DNA damage response 1 (REDD1) protein, a key mediator of stress, in autophagy-mediated NET formation inside the autophagy/NET/IL-1*β* signaling axis. These studies indicate that excessive release of NETs mediated by autophagy had a proinflammatory effect, which may also stimulate subsequent fibrosis [137, 138]. Therefore, autophagy and NETs may be potential therapeutic targets for human fibrotic diseases, but they play an important role in disrupting immune tolerance and inducing autoimmune disease as well [139]. This may explain the anti-inflammatory effects of drugs such as HCQ. However, HCQ side effects may also be a consequence of autophagy inhibition. Since autophagy is necessary in cell homeostasis, cardiac QT elongation [140, 141] and anaemia [142] are common CQ/HCQ side effects related to autophagy inhibition and need to be used with care. Additionally, CQ induces autophagy-independent effects, like Golgi disorganization [143] and pulmonary vasodilation [144], contributing to its controversial clinical activity. To avoid these effects and to obtain a limitation of autophagy in order to reduce cytokine activation, IL-6R mAb has been found effective in different settings [145, 146].

Dysfunction and dysregulation in autophagy are related to obesity [147–149], age, and male sex [150] in many diseases. In particular, obese people present an increased number of autophagosomes due to the combination of increased inflammation with inhibition of the last steps of autophagy [147, 148]. In elderly people, autophagy deficiency results in a reduced degradation of ROS-damaged proteins [151, 152], although oxidative stress is one of the most potent activators of this mechanism.

However, autophagy also has a protective role in reducing the excessive cytokine release in ARDS, representing a final stage in the regulation of severe inflammatory response. In fact, in a severe lung injury *in* vivo model, it has been demonstrated that the autophagy inducer rapamycin, but not inhibitors like CQ, is effective in reducing pulmonary oedema and increasing oxygenation [153]. Having a contrary action to CQ/HCQ use, also, mTOR inhibitors have been proposed as antiviral drugs in COVID-19 [154], but data on treated patients are limited and results from clinical trials are not available.

The autophagy role in SAR-CoV-2 infection needs to be experimentally established, and no data are yet available. Autophagy dysregulation based on previous coronaviruses data has been proposed as a hypothesis in SARS-CoV-2 infection [155], but neither conclusive data on CQ/HCQ use in SARS-CoV-2 infection nor data on other autophagy modulators are yet available.

## 6. SARS-CoV-2: Inflammation and Lung Disease

Inflammatory activation plays a pivotal role in the natural course of the SARS-CoV-2 infection. Immune system dysregulation is associated with the severity of SARS-CoV-2 infection, and severe cases display an increase of many cytokines: IL-1, IL-2, IL-6, IL-7, IL-8, IL-10, granulocyte colony stimulating factor (GSF), IFN-*γ*, inducible protein 10, monocyte chemoattractant protein 1, macrophage inflammatory protein 1-*α*, and TNF-*α* [8, 51]. Chinese data indicate high levels of ferritin and a low lymphocyte count as predictive of fatality [3, 8].

Lung biopsies reveal alveolar damage caused by immune cell infiltration, mostly macrophages and CD4 positive T lymphocytes, paralleled by a hyaline membrane and the proliferation of type II alveolar epithelia. Focal pulmonary fibrosis is associated with SARS-CoV-2 antigen in lung epithelia and macrophages [6]. Along with congested blood vessels, hyaline thrombi are often observed in microvessels [6]. SARS-CoV-2 is the first pandemic due to a coronavirus fatal lung infection. Previous coronaviruses (SARS-CoV and MERS-CoV) were equally lethal but did not spread worldwide like SARS-CoV-2. In previous SARS patients, hyperactivation of epidermal growth factor receptor (EGFR) signaling has been shown in response to acute lung injury [156]. In mice infected by SARS-CoV, Th2 are dysregulated as a consequence of an IFN production deficiency due to insufficient signal transducer and activator of transcription 1 (STAT1). These mice are prone to fibrosis because of the increase of EGFR followed by an increase in IL-8 and the recruitment of neutrophils [156]. In another *in vitro* study [157], it was seen that SARS-CoV infection could activate the chemokines of lung traffic, IL8 and IL17, through TLR9, which would cause symptoms and also monocyte-macrophage activation and coagulation upregulation. In a study on MERS-CoV, Aboagye et al. [158] showed that virus nucleocapsid causes an overexpression of antiviral genes, namely, TNF, IL6, IL8, and CXCL10, whose expression is stable over time. The pathogenesis of SARS-CoV-2 damage is still unknown, but unlike other SARS, SARS-CoV-2 shows an initial increase of the Th2 cytokines IL-4 and IL-10 which suppress inflammation [51] and balance the Th1 hyper-response.

**Figure 2:**
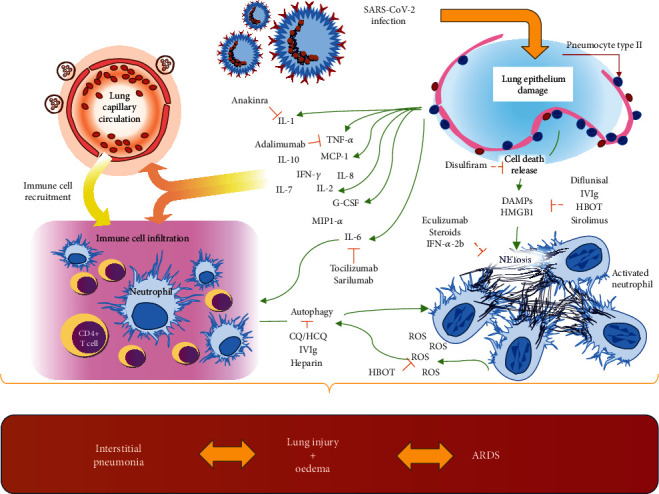
Infection of lung epithelium mediated by SARS-CoV-2 leads to cell release of multiple cytokines and DAMPs (HMGB1). These molecules induce immune cell recruitment as a direct action on resident cells and as a result of cytokine release in blood circulation. Activated neutrophils show increased autophagy and NETosis with the release of ROS. The resulting mechanism leads to lung injury and oedema resulting in interstitial pneumonia up to acute respiratory distress syndrome (ARDS). DAMPs: damage-associated molecular patterns; G-CSF: granulocyte colony stimulating factor; HMGB1: high-mobility group box 1; IL: interleukin; IFN-*γ*: interferon-*γ*; MCP1: monocyte chemoattractant protein 1; MIP1-*α*: macrophage inflammatory protein 1-*α*; NETosis: release of neutrophil extracellular traps; ROS: Reactive Oxygen Species; TNF-*α*: tumour necrosis factor-*α*.

In SARS-CoV-2 pathogenesis and lung damage, the immune response seems to be the main actor. However, it results in a double-edged action: while it firstly attempts to limit the viral infection, the continual SARS-CoV-2 stimuli turn it into a cytokine storm [159]. This corner point transforms less symptomatic disease into diffuse severe interstitial pneumonia [8, 51] (Figure 2). Cytokines play a role in the chemoattraction of immune cells. IL-8 has a central role in this mechanism, modulating the immune response promoting a disorientation in the migration of DCs and inducing NETosis [160, 161]. During viral respiratory infections, an increased level of IL-8 is present in airway secretions. This is positively correlated with neutrophil counts, neutrophil elastase levels, and clinical severity scores [162, 163]. The increase of IL-8 is one of the effects derived from HMGB1 stimulation of the respiratory epithelium and endothelium [164, 165]. Autophagy will enhance IL-8 release via NF-*κ*B activation [166]. At the same time, IL8 plays an important role in the activation of the PI3K/Akt/mTOR pathway [81], in a self-regulatory mechanism.

The immune host damage presents with different patterns. Lung and capillary damage is the main focus in COVID-19 [6, 12, 13, 90]. Pulmonary embolism may often complicate SARS-CoV-2 [167]. The microthrombi in small lung capillaries lead to small vessel vasculopathy through hyperperfusion of nonobstructed lung segments. A first report indicates cardiovascular disease as one of the first comorbidities [83]. Recent data also show acute coagulopathy and rheumatoid factor in patients who present with acute cardiac damage and an increased risk of mortality [168].

Endotheliitis appears to be a key factor in SARS-CoV-2 infection. Immune cell infiltration and consequent damage to the capillary walls lead to capillary leak, increased pulmonary oedema, activation of the coagulation cascade, and microthrombus formation. Varga et al. [13] found evidence of direct viral infection of the endothelial cells and diffuse endothelial inflammation. Although the virus uses the ACE2 receptor expressed by pneumocytes in the epithelial alveolar to infect the host, the ACE2 receptor is also widely expressed on endothelial cells. Immune cell response, by either direct viral infection of the endothelium or immune mediation, can result in widespread endothelial dysfunction associated with apoptosis, releasing numerous cytokines and DAMPs. The consequent activation of the coagulation cascade could be argued to lead to microthrombus formation. This phenomenon may be closely related to NETs. The NET-platelet interaction, as well as the activation of platelets mediated by the HMGB1-TLR4 axis, might influence the activation of the coagulation cascade and lead to diffuse microthrombus formation.

Recent data from COVID-19-recovered patients showed the presence of cellular immunity associated with humoral immunity. In fact, Ni et al. [169] found the production of antibodies specific for the S-protein binding domain in recovered patients and also described virus-neutralization activities in these recovered patients. This observation will be a cornerstone in future treatment of these patients, in terms of vaccines as well as acute treatment. The immune influence on the host damage occurring during SARS-CoV-2 infection is the milestone in the pathogenesis and evolution of COVID-19. The modulation of the immune response appears to be one of the fundamental strategies to treat COVID-19 patients.

## 7. Therapeutic Challenge for Inflammation Control in SARS-CoV-2

All data discussed are consistent with the possible rise of a cytokine storm syndrome in SARS-CoV-2. Indeed, the increase of IL-6 described in these patients [8] may open a new vista for promising therapy of severely ill patients. However, many immunomodulatory treatments have already been proposed and evaluated in clinical trials (Table 1 and Figure 2).

At present, oxygen and ventilation are necessary treatments in lung failure due to SARS-CoV-2 infection. Despite these mandatory treatments to ensure adequate tissue oxygenation, literature data indicate a possible role of the increasing NETosis [36, 170].

Indeed, considering previous coronavirus infections and *in vitro* data, some other immunomodulatory treatments proposed are Adalimumab, Anakinra, Diflunisal, Disulfiram, Baricitinib, Sirolimus, heparin, and intravenous immunoglobulin (IVIg) (Table 1), as well as steroids and INF, whose efficacy is being evaluated in clinical trials [171–174].

### 7.1. Remdesivir

Remdesivir (RDV) is one of the antiviral drugs used in SARS-CoV-2 infection. RDV is an adenosine analogue that induces premature or delayed termination of the viral RNA chains. First developed to treat Ebola infection [175], it was considered a promising antiviral drug against a broad range of RNA viruses including coronaviruses. The drug acts by inhibiting the key enzyme RNA-dependent RNA polymerase (RdRP) after the virus enters target cells [176]. When incorporated into a specific position in the RNA chain, RDV causes the inhibition of RNA synthesis at five nucleotides down to the site of the drug incorporation, thus delaying chain termination [176]. Based on previous coronavirus infection data, RDV improves disease outcomes and attenuates viral loads in MERS-CoV-infected mice with a critical inflammatory response. RDV also exhibits protective effects against acute lung injury (ALI) in rodent animals, by reducing neutrophil infiltration, which was associated with the mediation of IFNs [177]. Studies in nonhuman primates have shown the prophylactic and therapeutic value of the drug in other coronavirus infections like MERS-CoV [178]. Another recent *in vitro* study reported a role for RDV in SARS-CoV-2 infection. Investigators concluded that among several drugs tested, only RDV associated with CQ could inhibit the virus infection in human cells sensitive to SARS-CoV-2 [179]. Recently, the U.S. Food and Drug Administration authorized Remdesivir, on the 1st of May 2020, for treatment in COVID-19 patients. It is currently the only drug approved to treat SARS-CoV-2 infections. RDV is still under investigation, but preliminary data indicate an increase in survival of COVID-19 patients treated with this drug. The main S3 and S4 side effects were anaemia or acute kidney injury. Despite data proving Remdesivir efficacy in reducing SARS-CoV-2 spread in COVID-19 patients, no evidence has been found that it can control the immune reaction. It could be speculated that the reduced SARS-CoV-2 load influences the formation of NETs or DAMPs, but no data are yet available. In fact, as we point out in this review, NETosis and DAMPs appear to be two promising treatment targets. In this view, to obtain a better control of inflammation in COVID-19 patients, it should be necessary to add drugs that will act on the immune system.

### 7.2. Chloroquine/Hydroxychloroquine

One of the treatments most widely used in SARS-CoV-2 is the antimalarial drug chloroquine (CQ)/hydroxychloroquine (HCQ). This drug has multiple functions. The best known is its ability to alkalise the phagolysosome, one of the mechanisms underlying its antiviral mechanism [180]. In sepsis, the role of CQ in cell death reduction has been demonstrated, preventing the release of HMGB1. In particular, an immunomodulatory action has been proposed in SARS infection [180, 181]. IL-2 is increased in SARS-CoV-2 and plays a crucial role in “priming” T-cells for Th2 differentiation [8, 182]. CQ inhibits T-cell proliferation by reducing IL-2 production and responsiveness [183]. The Th2 response may play a role in suppressing inflammation in SARS-CoV-2 infection, and CQ/HCQ could have an impact on the immune response to the virus. Nevertheless, considering its anti-inflammatory properties, CQ/HCQ may have some effect on SARS [184], especially by inhibiting the production of proinflammatory cytokines (TNF*α*, IL-6), hence blocking the cascade of pathways which lead to ARDS [184]. Initial reports indicate clinical benefit following CQ/HCQ use. However, CQ/HCQ has been found to have a dual role in sepsis. Caution has been suggested [185] because no high-quality clinical data or results of clinical trials showing a clear benefit of these agents for COVID-19 have been reported [186]. While it prevents lethal cases in a sepsis *in vivo* model [187], via preventing autophagy, the same effect in severe cases leads to a worsening of disease [153]. This dual role should be related to prolonged hypoxia. In fact, literature data indicate that during the early phase of hypoxia, CQ induces a reduction of autophagy, HMGB1, IL-6, and organ damage, while in a prolonged hypoxic state, it favours further damage, enhancing the cellular damage via apoptosis and autophagy [188]. However, considering the role of T-cell immunity in the humoral response to SARS-CoV-2 infection [169], CQ/HCQ could play a detrimental role on the formation of protecting immunoglobulins.

At the time of this review, the standardized dose regimen is CQ 500 mg b.i.d. or HCQ 200 mg b.i.d [189]. but several clinical trials on CQ and HCQ-based regimens for SARS-CoV-2 are still ongoing (Table 1) [173] and such use is still off-label.

Yao et al. [190] report *in vitro* activity of HCQ in inhibiting SARS-CoV-2. Open-label data indicate a clinical benefit in patients treated with CQ/HCQ [191]. Due to the above evidence, the negligible cost, and the wide use worldwide, CQ/HCQ has been considered a potentially useful drug in nonsevere patients affected by SARS-CoV-2 [190–193]. CQ has been demonstrated to be an enhancer of zinc cell uptake in a concentration-dependent manner, acting as a zinc ionophore [194]. Zinc inhibits RNA-dependent RNA polymerase and has been shown to do this in vitro against previous SARS-CoV [195]. In a recent preprint, Carlucci et al. [196] demonstrated that patients experienced a reduced mortality, disease severity, and need of ICU or ventilation when they underwent zinc treatment in addition to standard HCQ administration. In preliminary data from a double-blind, randomized clinical trial on mild COVID-19, HCQ administration appears to be effective in preventing the mild-to-severe transition [197].

However, contrasting data have been published. Recently, Rosenberg et al. had described an increased mortality in patients in hospitalized COVID-19 patients who underwent HCQ treatment compared to Azithromycin treatment [198]. Moreover, there was a greater mortality among patients who took HCQ and Azithromycin than other treatments [198]. The main death cause in these patients was cardiac arrest [198], probably due to elongation of the QT interval [195] shown at electrocardiogram, a common side effect of HCQ. Recent data have been discussed and retracted in the international literature [199] suggesting that evidence-based and randomized data are needed. Clinical trial results should reveal definitive indications for CQ/HCQ use.

### 7.3. Tocilizumab and Other IL-6R Inhibitors

The first IL-6 receptor- (IL-6R-) neutralizing mAb, called Tocilizumab, has been approved in more than 100 countries for the treatment of autoimmune diseases [200]. Blockade of the IL-6 cascade could have a rationale to modulate the inflammatory response. Tocilizumab was experimentally administered intravenously in the treatment of SARS-CoV-2 with encouraging results [201].

Preliminary data indicate Tocilizumab efficacy. The first Chinese report on the drug used in severe patients had described a reduction of temperature and CRP associated with an increase in SaO_2_% and lymphocyte percentage, resulting in a better clinical outcome [202]. Also, Italian off-label use in hospitalized patients indicates that Tocilizumab exerts a rapidly beneficial effect on fever and inflammatory markers, inducing a dramatic drop of body temperature and CRP value, a significant increase in lymphocyte count, and a benefit in clinically severe disease [203, 204].

However, hyperglycaemia may influence IL-6R inhibitors. In fact, recent data on hyperglycaemic vs. normoglycaemic patients found fivefold higher IL-6 levels during hyperglycaemia [205]. Moreover, in hyperglycaemic patients, higher IL-6 plasma levels reduced the effects of TCZ [205], indicating that optimal COVID-19 infection management with TCZ is not achieved during hyperglycaemia in either diabetic or nondiabetic patients.

Based on these recent off-label data in the Chinese and Italian populations, many trials worldwide of Tocilizumab as well as other IL-6R inhibitors have been started (Table 1).

### 7.4. Sirolimus

Sirolimus is the commercial drug form of rapamycin. It blocks mTOR and subsequent pathways, reducing cytokine (such as IL-6 and TNF-*α*) expression. It enhances NETosis even without external stimuli, reducing the expression of HMGB1 and stimulating autophagy as a direct effect of mTOR blockade. The mTOR pathway functions as a central regulator of cell metabolism, growth, proliferation, and survival. It senses both intracellular and extracellular signals to control protein synthesis, lipid metabolism, autophagy, and transcription [206]. mTOR is a protein kinase part of the compound of two distinct multiprotein complexes, mTOR complex 1 (mTORC1) and mTOR complex 2 (mTORC2). They sense different signals to control different cellular processes. mTORC1 mainly functions as a nutrient/energy/redox sensor and controls protein synthesis, lipid metabolism, and organelle biogenesis; it is rapamycin-sensitive [206]. On the contrary, rapamycin- insensitive mTORC2 serves as a regulator of the actin cytoskeleton, metabolism, and cell survival [206]. mTOR also controls the gene expression of myeloid immune cells to regulate their migration and cytokine expression [207]. In addition, the mTOR pathway also plays a vital role in B-cell development in the germinal center [208]. Due to these pathways, pathogens have evolved strategies to target this pathway within DCs and macrophages, promoting immune escape [40]. Inhibition of mTORC1 enhances the T-cell stimulatory activity of dendritic cells (DCs) and promotes the autophagy of macrophages. mTOR inhibitors also reduce antigen-specific memory B-cells after B-cell activation [207]. Thus, in high-risk patients at an early stage of SARS-CoV-2 infection, we speculate that the immune enhancement process can be avoided and severe symptoms can be alleviated. The mTOR inhibitor rapamycin also enhances the magnitude and quality of viral specific CD8+ T-cell responses to vaccination in macaques [209]. These studies have elucidated new mechanistic characteristics of mTOR inhibitors and suggest immune applications beyond their role as immunosuppressants. On the other hand, an *in vitro* study demonstrated that the mTOR inhibitor rapamycin blocks the replication of MERS-CoV [210]. Adjuvant treatment with mTOR inhibitors and corticosteroids can significantly improve the outcome in ICU patients infected with the H1N1 influenza virus [210]. Currently, two clinical trials using the mTOR inhibitor Sirolimus to treat COVID-19 patients are ongoing.

### 7.5. Steroids

Corticosteroids are well-known drugs for the treatment of inflammation and autoimmune diseases. They bind to nuclear receptors to reduce the release of proinflammatory cytokines. Steroids induce a reduction of NET formation in *in vitro* and *vivo* models [211]. Steroids also reduce the release of HMGB1 and its interaction with TLR4 [212] and modulate autophagy, inhibiting apoptosis and enhancing regulatory autophagosomes [212, 213].

However, in SARS-CoV-2 infection, steroid treatment has not shown clear results in the literature and there is no indication statement.

The use of corticosteroids in patients presenting with ARDS of different aetiologies remains controversial. Animal experiments provide evidence for the use of glucocorticoids during the acute phase of severe disease to reduce inflammation, attenuate ALI, and improve survival [214]. Results in the literature, mainly derived from observational studies, were nonconclusive and sometimes conflicting. Globally, high-dose glucocorticoids are among the drugs most frequently used in ARDS [215]. Systemic corticosteroids have long been used in critically ill patients presenting with ARDS, given their role in lowering the circulating levels of proinflammatory mediators [216, 217]. Moreover, adequate, prolonged glucocorticoid supplementation has proved to mitigate the endogenous corticosteroid insufficiency, thus enhancing the resolution of lung and systemic inflammation [218]. One systematic review found that, compared with placebo, prolonged glucocorticoid treatment improved clinical outcomes [214]. A recent meta-analysis combined four RCTs evaluating prolonged methylprednisolone therapy for ARDS and reported a significant reduction in mortality, with an increase in ventilator-free days (13 vs. 7, *p* < 0.001) [219]. However, other studies have failed to provide convincing evidence of the efficacy of corticosteroids in decreasing the mortality of ARDS, suggesting that glucocorticoid therapy is not necessary in this condition and may even aggravate the clinical course of the disease.

Steroid use is still being debated also for the previous coronavirus infections, SARS and MERS.

Corticosteroids therapy was used in the treatment of severe SARS, supported by early anecdotal experience [220, 221]. In March 2003, China suggested that high-dose glucocorticoids should be used if patients affected by SARS had fever persisting for more than 3 days or if radiologic findings were suggestive of persistent lung involvement or progressive deterioration [222].

One systematic review on SARS-CoV infection found that 25 studies were inconclusive regarding the role of the use of glucocorticoids in addition to standard therapy, while four studies demonstrated that systemic glucocorticoids in SARS patients may cause possible harm [223].

Glucocorticoid therapy was also used for critical MERS patients. Steroids were given in hypoxaemic patients with MERS-CoV pneumonia who were not showing signs of improvement [224]. The study reported that there was no difference in 90-day mortality, and in these patients, there was associated delayed MERS-CoV RNA clearance.

Recent evidence suggests that a subset of patients with severe COVID-19 may have the cytokine storm syndrome [51], which is a condition frequently related to lung involvement (including ARDS) [121] and multiorgan failure. In order to induce immunosuppression to antagonize virus-driven hyperinflammation, treatments with Tocilizumab are ongoing in patients in whom a hypercytokinemia laboratory pattern is identified. In these patients, a therapeutic role can also be hypothesized for corticosteroids [225].

Data on their use in COVID-19 are still not conclusive. In a systematic review including 542 Chinese patients, the authors did not find definitive evidence for nonsteroid use in SARS-CoV-2 infection [226]. In particular, two studies reported negative findings regarding these medications [227, 228]; one reported no significant association between corticosteroids and clinical outcomes [229], while the other concluded that they reduce mortality in patients with COVID-19 pneumonia developing ARDS [230]. Recent data also indicate that early, short course methylprednisolone is effective to reduce mortality and progression to respiratory failure, ARDS and ICU admission [231]. Another study on SARS-CoV-2 pneumonia in Wuhan had showed a reduced length of ICU hospitalization associated to an early peripheral oxygen recovery in patients treated with iv methylprednisolone [232]. From these data, it is possible to argue that early short-term administration of methylprednisolone was associated with better clinical outcomes in patients with severe COVID-19 pneumonia and should be considered before the occurrence of ARDS.

### 7.6. Heparins

Heparin is a heterogeneous mixture of branched glycosaminoglycans defined as Unfractionated Heparin (UFH). Binding the enzyme inhibitor antithrombin III (AT), it causes a conformational change, activation, and consequently the inactivation of thrombin, factor Xa, and other proteases involved in the coagulation cascade [233]. The same effect is mediated by low molecular weight molecule heparin (LMWHs). These are polysulphated glycosaminoglycans that are about one-third the molecular weight of UFH [233]. Both have a possible role in immune modulation. LMWH has been found to reduce NETosis due to a reduction of neutrophil activation autophagy [135]. Modulation of neutrophil activation is also a result of the inhibition of HMGB1 binding to the immune cell surface, making neutrophil refractory to DAMP stimulation [84, 135].

Heparin appears to be one of the most useful therapies in SARS-CoV-2 infection. Analyzing previous SARS-CoV data, it has been found that coagulation factor Xa is one of the coronavirus proteases used to enter into host cells [234]. Moreover, activation of the coagulation cascade is one of the main frames of COVID-19 [90] and possibly results in NETosis and DAMP release. From this perspective, heparin appears useful in SARS-CoV-2-infected patients. To check coagulation activation, D-dimers were proposed as markers. Anticoagulation therapy is recommended for COVID-19 patients when high D-dimer levels are detected [235] or in immobilized hospitalized patients [236], except those in whom anticoagulants are contraindicated. However, it has to be taken into account that D-dimers are specific markers and their increase could be also related to the inflammation itself.

Tang et al. [237] reported a major improvement of clot activation markers and a reduction of 28-day mortality in COVID-19 patients treated with heparin. The recommended therapeutic dose of LMWH is 100 U per kg weight per 12 h by subcutaneous injection for at least 5 days, while prophylactic LMWH has to be considered when multiple risk factors are detected [236].

### 7.7. Convalescent Plasma and Intravenous Immunoglobulins

The infusion of plasma serum obtained from PCR-negative, recovered patients, containing IgG anti-SARS-CoV-2 (hyperimmune IgG-containing plasma (HIgCP)), is a therapeutic approach in newly infected subjects, based on previous experiences related to other viral infections, like SARS-CoV, MERS-CoV, Ebola, H5N1 avian influenza, and H1N1 influenza [238–241].

Similarly, intravenous immunoglobulins (IVIg) had a role in the modulation of immune response. The anti-inflammatory/immune-regulatory role of IVIg also relies on their Fc region interaction with the corresponding Fc*γ* receptors (Fc*γ*Rs). Fc*γ*Rs are expressed on cells involved in natural (phagocytes) and adaptive (T-cells, B-cells) immunity and on antigen-presenting cells, necessary to bridge natural and adaptive immunity. The interaction may modulate signaling through Fc*γ*Rs, ultimately inducing potent anti-inflammatory effects [242, 243]. IVIg may also influence the number and function of Tregs which help to control inflammation and inhibit T-cell activation [244], TNF-*α* production, and IL-6 and matrix metalloproteinase 9 activity. IVIg also reduces NET formation [245] and may have a role in modulating a regulatory autophagy. Moreover, Fc fragment and IVIg may reduce cytokine and DAMP production [246, 247] and protect against HMGB1-induced cell death, modulating TLR and RAGE expression [248].

These properties are the rationale for suggesting HIgCP and IVIg use in SARS-CoV-2 infection to prevent and counteract the cytokine-mediated interstitial and alveolar wall oedema responsible for ARDS.

The administration of HIgCP could be useful to treat or prevent SARS-CoV-2-induced ARDS. The administration of Ig may accelerate virus clearance, also considering the finding of neutralizing Ig in SARS-CoV-2-recovered patients [169, 249]. However, the need for a blood group match between donor and recipient, as well as the risk for other viral infections, makes HIgCP less suitable than IVIg for large-scale administration. Preliminary clinical experiences are promising [250], but are far from conclusive. Ongoing clinical trials are assessing the true effectiveness of IVIg in COVID-19 patients.

### 7.8. Interferons

Interferon is a recombinant cytokine with antiviral properties [251]. It is administered as a single agent or in combination with ribavirin, a guanine derivative that induces lethal mutations in RNA-dependent replication in RNA viruses.

Type I IFN has been demonstrated to enhance NETosis. The pathway presents a two-way mechanism. *In vitro*, NETs trigger TLR on DCs, resulting in the production of interferon alpha (IFN*α*) [252, 253]. In turn, IFN*α* primes neutrophils to release NETs [252, 253], resulting in a perpetuating pathogenic cycle of NET release and IFN*α* production. HMGB1 is related to IFN*α* in sustaining inflammation. In particular, IFN is effective in promoting HMGB1 release in the bloodstream during gram-negative infection [254]. Both HMGB1 and IFN are related to the release of inflammatory cytokines, especially during hypoxia [255], and concur in sustaining inflammation via the TLR4/MyD88/NF-*κ*B inflammatory signal pathway [256, 257]. IFN has a role also in modulating autophagy, necessary for viral cleavage [258]. This is one of the antiviral properties of IFN. In fact, viral replication is an autophagy-dependent mechanism demonstrated in respiratory viruses [259]. The modulation of autophagy interferes with replication, facilitating viral cleavage [258, 259].

Conflicting data were reported on the role of interferon in previous coronavirus diseases. It was found to be effective in SARS-CoV and also tested in combination with corticosteroids [260]. Interferon shows an antiviral activity, by binding to interferon receptor type 1. Furthermore, it promotes dimerization and activates Janus kinase 1 (Jak1) and tyrosine kinase 2 (Tyk2) phosphorylation. STAT1 and STAT2 bind to the phosphorylated IFN receptor and trigger the expression of immunomodulators and antiviral protein expression including protein kinase R (PKR) [261].

In a retrospective observational study of 32 MERS patients, mortality with interferon *α*2a was 85% versus 64% with interferon *β*1a [262]. In a multicenter observational study of 349 critically ill MERS patients, the interferon and ribavirin combination was not associated with any benefit on mortality or viral clearance [263]. There are multiple reports of the antiviral activity of IFN against coronaviruses, and these agents might also be effective against SARS-CoV-2. At present, there is no evidence to support the effectiveness of interferon for COVID-19. Although many reports indicate its use in China [264–267], the data are far from conclusive and results from clinical trials are needed. Additional clinical studies are required to approve this drug for SARS-CoV-2 therapy.

### 7.9. Other Treatments

Anakinra is a modified human IL-1 receptor antagonist (IL-1RA) approved for use in RA patients. The IL-1 family of receptors triggers the innate immune response and was associated with damaging inflammation [268]. NETosis is one of the immune pathways involved in the stimulation of IL-1 release [269]. The increase in IL-1 induces a consequent increase in IL-8, enhancing NET formation in a self-perpetuating loop [269]. Due to this, IL-1RA has a role in blocking NET formation [269]. Anti-IL1R may have a role in blocking DAMP release. In fact, IL-1*α* is related to an HMGB1 increase in a respiratory inflammation model, and IL-1RA is effective to reduce HMGB1 in bronchoalveolar lavage fluid [270]. Moreover, anti-IL1R may restore regulatory autophagy [271, 272]. Due to its mechanism of action, mitigation of the cytokine storm could be hypothesized. Clinical trials involving Anakinra treatment are ongoing, some of them using Tocilizumab for comparison. Excluding anecdotal cases [273, 274], no clinical use nor preliminary data are available for SARS-CoV-2.

Eculizumab is a humanized IgG mAb that binds to complement protein C5 and prevents the formation of membrane attack complex (MAC). It is able to block NET formation due to complement activation [275]. In COVID-19, it is under investigation in a couple of randomized clinical trials. Preliminary reports indicate that it was effective to salvage COVID-19 patients admitted to the intensive care unit with severe pneumonia or ARDS [276].

Hyperbaric oxygen therapy (HBOT) is a medical use of oxygen at an ambient pressure higher than atmospheric pressure. It reduces inflammation, modulating cytokine release, and increases ROS production, reduces apoptosis, and modulates leukocyte activation and adhesion [277]. This treatment appears effective to reduce ROS-dependent NETs release [278], autophagy [279] and HMGB1 pathway activation [280]. In a small case series of COVID-19 patients, HBOT appears effective to reduce the respiratory rate and improve blood oxygenation 24 hours after treatment [281]. However, it was given for compassionate use and a larger prospective clinical trial is needed.

## 8. Conclusions

The SARS-CoV-2 infection leads to severe disease, and no evidence-based data are yet available to support the best treatment choice. The immune system is the main counterpart in aggravating the disease and host damage. NETs could be a promising target to prevent lung injury progression. At the same time, HMGB1 should be the key point targeted for both the prevention of progression and lung injury treatment. Further evidence is needed with large multicentric clinical trials.

## Figures and Tables

**Table 1:** immunomodulatory drugs in clinical development to treat SARS-CoV-2. This table lists agents under investigation and/or theoretically being considered for patient management. At this time, no recommendation can be made for any of these agents and some of them are off-label use.

Drug or cocktail	Status and mechanisms	NETs	DAMPs	Autophagy	Clinical trials (trial posting date)
Adalimumab	Anti-TNF-*α* humanized mAb able to suppress the physiologic response to TNF-*α*, which is part of the inflammatory response in autoimmune and immune-mediated disorders	No data	No data	In autoimmune disease, it inhibits autophagy, enhancing regulation	One clinical trial enrolled in China (ChiCTR2000030089) in combination with standard treatment in severe disease
Anakinra	IL-1 receptor antagonist hypothesized to quell cytokine storming. No data available as adjunctive therapy for SARS-CoV-2	Possible role in blocking NET formation	Anti-IL1R may have a role in blocking DAMP release	Anti-IL1R may have a role in restoring a regulatory function	12 clinical trials as single drug or in combination are enrolling patients in Europe
Bevacizumab	Recombinant humanized mAb that prevents vascular endothelial growth factor (VEGF) association with endothelial receptors Flt-1 and KDR approved for multiple cancers in the US	No data	No activity	Autophagy promotes resistance to Bevacizumab	Being evaluated in two clinical trials in China for COVID-19 (NCT04305106 and NCT04275414) and one international trial (NCT04344782), no data are currently available to support its use
Chloroquine or hydroxychloroquine	Endosomal acidification fusion inhibitor	Inhibition of NETosis as a consequence of autophagy blockade	Inhibition of HMGB1 activity in a septic *in vivo* model	Blocks autophagosome fusion and degradation	At least 300 trials, alone and in combination, for mild symptoms and prophylaxis, have been registered with the FDA and Chinese clinical trials
Convalescent plasma	Plasma from convalescent patients who have recovered from the SARS-CoV-2 infection has been used with success much in the same way as for SARS-CoV-1, MERS, Ebola, and H1N1 influenza	Possible role overlapping IVIg	Possible role overlapping IVIg	Possible role overlapping IVIg	Up to 250 clinical trials enrolled patients with severe SARS-CoV-2 disease in the US, China, Italy, and Mexico registered with the FDA and Chinese clinical trials
Diflunisal	Diflunisal, a nonsteroid anti-inflammatory drug (NSAID) included in the salicylate class, has a specific indication to prevent flogosis and pain control in joint disease	No data	Blocks immune cell recruitment inhibiting HMGB1/CXCL12 activation	No data	No clinical trial for SARS-CoV-2
Disulfiram	Thiuram derivative that blocks alcohol oxidation, demonstrating ability to competitively inhibit the papain-like proteases of SARS but no clinical data. No *in vitro* or clinical data available for SARS-CoV-2	No data	Reduces the release of DAMPs in apoptotic cells	Dual role in Disulfiram mechanism studied in cancer	None
Eculizumab	Humanized IgG mAb that binds to complement protein C5 and prevents formation of membrane attack complex (MAC)	Blocking NET formation via complement activation	No data	No data	Being evaluated in a clinical trial (NCT04288713) for SARS-CoV-2 to quell immune response. New clinical trial (NCT04346797) is recruited as a single agent
Heparin/low molecular weight heparin	Heparin binds to the enzyme inhibitor antithrombin III (AT), causing a conformational change that results in activation and consequent inactivation of thrombin, factor Xa, and other proteases. Possible role in immune modulation	Reduction of NETosis due to a reduction of autophagy	Inhibition of activity via blockade of HMGB1 binding to immune cell surface	Reduction of neutrophil activation autophagy	At least 40 clinical trials planned to evaluate its efficacy in the prevention of SARS-CoV-2 complications
Hyperbaric oxygen	Hyperbaric oxygen therapy is a medical use of oxygen at an ambient pressure higher than atmospheric pressure. It reduces inflammation, modulating cytokine release, and increases ROS production, reduces apoptosis, and modulates leukocyte activation and adhesion	Reduction of ROS-dependentNET release	Reduced activation of HMGB1 pathways	Inhibits autophagy activation reducing ROS	8 clinical trials are ongoing to evaluate its efficacy in ARDS and pneumonia
Interferon alpha-2b alone or in combination with other drugs	Interferon *α*-2b is a recombinant cytokine with antiviral properties; ribavirin is a guanine derivative; as above	Type I IFN enhance NETosis	HMGB1 is related to IFN*α* in sustaining inflammation	Possible role in enhancing antiviral autophagy	60 clinical trials registered as a single agent or in combination for SARS-CoV-2
Intravenous immunoglobulin (IVIg)	IVIg remain on critical national shortage in the US. The benefit in patients with SARS-CoV-2 is unclear	IVIg reduce NET formation	Protection against HMGB1-induced cell death modulating TLR and RAGE expressions	IVIg may have a role in modulating a regulatory function	More than 250 clinical trials are planned all over the world to evaluate efficacy in SARS-CoV-2 pneumonia
Methylprednisolone	Synthetic corticosteroid that binds to nuclear receptors to dampen proinflammatory cytokines	Reduction of NET formation in *in vitro* and *in vivo* model	Reduces the release of HMGB1 and its interaction with TLR4	Modulation of regulatory autophagy	25 clinical trials in COVID-19 disease as a single agent or in combination with standard care or mAb in US and China
Remdesivir	Adenosine analogue that leads to premature or delayed termination of the viral RNA chains	No data	No data	No data	First approved specifically to treat COVID-19
Sarilumab	IL-6 receptor antagonist FDA-approved for rheumatoid arthritis	Possible role in blocking NET formation	No data	Role in blocking regulatory autophagy	15 clinical trials in SARS-CoV-2 disease as a single agent or in combination
Sirolimus	Commercial drug form of rapamycin blocks mTOR and subsequent pathways, reducing cytokine (such as IL-6 and TNF-*α*) expression	Enhances NETosis also without external stimuli	Reduces the expression of HMGB1	Stimulation of autophagy as a direct effect of mTOR blockade	4 clinical trials as a single agent, enrolling patients affected by SARS-CoV-2 disease
Tocilizumab	Humanized mAb targeting IL-6	Possible role in blocking NET formation	No data	Role in blocking hypoxia-induced autophagy	Up to 60 clinical trials in different countries

Last search run on June 06 using https://clinicaltrials.gov and http://www.chictr.org.cn. This table lists agents being investigated and/or theoretically considered for the management of SARS-CoV-2-infected patients. At this time, no recommendation can be made for any of these agents. In general, they should be avoided without additional supporting evidence.

## Abbreviations

ARDS: Acute respiratory distress syndrome
Baf-A1: Bafilomycin A1
CQ: Chloroquine
COVID-19: Coronavirus Disease 19
CPAP: Continuous positive airway pressure
CRP: C-reactive protein
CXCL12: CXC chemokine ligand 12
CXCR4: CXC chemokine receptor type 4
DAMPs: Damage-associated molecular patterns
DCs: Dendritic cells
ds-HMGB1: Disulphide bond HMGB1
EGFR: Epidermal growth factor receptor
fr-HMGB1: Reduced form HMGB1
G-CSF: Granulocyte-colony stimulating factor
HCQ: Hydroxychloroquine
HIF-1*α*: Hypoxia-inducible factor 1*α*
HMGB1: High-mobility group box 1
IL: Interleukin
HSPs: Heat shock proteins
IFN: Interferon
IVIg: Intravenous immunoglobulin
mAb: Monoclonal antibody
MAPKs: Mitogen-activated protein kinases
MCP: Monocyte chemoattractant protein
MERS-CoV-2: Middle East Respiratory Syndrome Coronavirus type 2
MIP: Macrophage inflammatory protein
mTOR: Mammalian target of rapamycin
NETs: Neutrophil extracellular traps
NF-*κ*B: Nuclear factor of kappa light polypeptide gene enhancer in B-cells
PAD4: Protein Arginine Deaminase 4
PCT: Procalcitonin
PKC: Protein kinase C
PKR: Protein kinase R
PMA: Mitogen phorbol myristate acetate
polyP: Polyphosphates
RAGE: Receptor for advanced glycation end products
RDV: Remdesivir
REDD1: Regulated in development and DNA damage response 1
ROS: Reactive Oxygen Species
RSV: Syncytial respiratory virus
SARS: Severe Acute Respiratory Syndrome
SARS-CoV: Severe Acute Respiratory Syndrome Coronavirus type 1
SARS-CoV-2: Severe Acute Respiratory Syndrome Coronavirus type 2
STAT1: Signal transducer and activator of transcription 1
Th: T helper lymphocytes
TLR: Toll-like receptor
TNF: Tumour necrosis factor
Tregs: Regulatory T-cells.
